# Radical versus Local Surgical Excision for Early Rectal Cancer: A Systematic Review and Meta-Analysis

**DOI:** 10.26502/aimr.0160

**Published:** 2024-01-20

**Authors:** Sarah El-Nakeep, Samragnyi Madala, Anusha Chidharla, Balarama Krishna Surapaneni, Subhrajit Saha, Benjamin Martin, Anup Kasi

**Affiliations:** 1 Faculty of Medicine, Ain Shams University, Cairo, Egypt; 2 University of Iowa Hospitals and Clinics, 200 Hawkins Drive, Iowa City, IA, United States; 52242; 3 Division of Colorectal Surgery, University of Kansas Medical Center, 3901 Rainbow Blvd, Kansas City, KS, United States: 66160; 4 Division of Radiation Oncology, University of Kansas Medical Center, 3901 Rainbow Blvd, Kansas City, KS, United States: 66160; 5 Division of Medical Oncology, University of Kansas Cancer Center, 2650 Shawnee Mission Pkwy, Westwood, KS, United States 66205

**Keywords:** Early rectal cancer, T1-T2 rectal cancer, Metaanalysis, Radical excision, Local excision

## Abstract

**Background:**

Radical excision (RE) for rectal cancer carries a higher risk of mortality and morbidity, while local excision (LE) could decrease these postoperative risks. However, the long-term benefit of LE is still debatable.

**Aim:**

To study the effectiveness of LE versus RE in T1 and T2 rectal cancer.

**Methods:**

A systematic review and meta-analysis was conducted using key databases like PubMed and ClinicalTrials.gov. Only cohort studies and randomized controlled trials were included. RevMan 5.4 tool was used for data analysis. Both clinical and statistical heterogeneity of the studies were assessed, and I^2^ >75% was considered as highly heterogeneous. The primary outcomes being measured were 5-year overall survival (OS) and 5-year disease free survival (DFS). A subgroup analysis of patients with T1-only was also conducted, without adjuvant chemo/radiotherapy.

**Results:**

A total of 18 studies were included for final meta-analysis. Four were RCTs, while the other 15 were retrospective cohort studies. One included study had data from both RCT and non-RCT study groups. Nine studies were multicentered or national studies while nine were unicentral.

There was no difference in risk ratio (RR) between OS: RR 0.95, 95% Confidence Interval (CI) [0.91, 0.99] and DFS: RR 0.93, 95% CI [0.87, 1.01]. There were lower hazards ratios in OS: RR 1.41, 95% CI [1.14, 1.74] and DFS: RR 1.95, 95% CI [1.36, 2.78] with radical, as compared to LE. Lower recurrence rate was associated with RE. Random effect model was used due to clinical heterogeneity between studies (different surgical procedures, tumor staging, adjuvant chemo or radiotherapy).

**Conclusions:**

LE for early-stage rectal cancer has lower 5-year OS and DFS than RE, with higher local recurrence rate. However, LE is associated with lower early postoperative mortality, morbidity and length of stay as compared to RE.

## Introduction

Colorectal cancer (CRC) is the third most common malignancy in the United States. Rectal cancer occurs in 40,000 people annually in the United States with the incidence rising among the younger population [1].

Rectal cancer is associated with a higher overall risk of recurrence than colon cancer with a similar stage. The local recurrence in rectal cancer is believed to be secondary to the anatomic location and inherent challenge for surgeons to achieve clear, circumferential margins. The standard of care surgery for mid-to-low rectal cancers currently is total mesorectal excision (TME) with RE of the rectum and the draining lymph nodes. This encompasses the surgical techniques of low anterior resection (LAR) and abdominoperineal resection (APR). For select patients LE can be done via transanal excision, transanal endoscopic microsurgery (TEM), or transanal minimally invasive surgery (TAMIS) [2].

LE is an organ preserving surgery compared to RE with TME for patients with stage I rectal cancer. Data regarding local recurrence and long term patient outcomes for patients who underwent LE for T2 tumors is limited [3]. The long-term oncologic outcomes of LE are still debatable. Currently there is no standard treatment consensus for early rectal cancers and the best standard of care treatment for early rectal cancers has yet to be defined. Therefore, we aim to do a systematic review and meta-analysis to study the effect of LE versus RE in T1 and T2 rectal cancer.

Radical excision has a higher risk of morbidity and mortality, while LE could reduce the postoperative complications. Generally, stage II and III rectal cancers are treated with trimodality therapy with chemotherapy, radiation therapy (RT) and surgery unlike colon cancer [4]. The determination of an optimal treatment plan for a rectal cancer patient is complex. Additionally, consideration should be given to the postoperative complications after surgery like restoring and maintaining bowel function, preservation of genitourinary functions and anal incontinence, which can affect quality of life.

Risk of local recurrence is higher in patients with rectal cancer compared to colon cancer. Treatment decision should be made carefully in each patient with regards to use of multimodality therapy involving chemotherapy, radiation treatment, and surgery. For early-stage rectal cancers local excision is attractive because of sphincter preservation and decreased morbidity. However, there is some contradictory data in the literature about local recurrence and overall survival when compared to radical excision [5].

## Methods:

We conducted the search on 15 June 2021 (from inception until 15 June 2021), using the following keywords and PICOTS as shown in the table.

We searched PubMed, CENTRAL, and ClinicalTrials.gov databases, using relevant keywords. We included only cohort studies and randomized controlled trials.

All included trials contained at least one of the primary outcomes.

### Data analysis:

We collected data for our predesigned primary outcomes:

1. Overall survival at 5 years (RR and HR)
2. Disease free survival after 5 years (RR and HR)
3. Local recurrence rate
4. Total postoperative 30-days mortality
5. Post operative hospital stay in days - postoperative
6. All adverse events

### Statistical calculation:

We used Revman desktop version 5.4 for data analysis. For continuous outcomes we used standardized mean difference (SMD), the only continuous outcome data were for hospital stay in days- postoperative. We used standardized mean difference, as the data presented in the studies as mean and standard deviation, or interquartile range. In case of IQR we used the median as mean, and divided IQR/1.35.

For survival data outcomes we calculated both the risk ratio (RR) and hazard ratio (HR). We calculated both hazards ratio (HR) and risk ratio (RR) for the 5-years survival analyses, with their 95% confidence intervals (CI). We extracted HR from the studies if mentioned, or else from Kaplan Meyer’s curves using the methods presented in Tierney et al [6].

We assessed both clinical and statistical heterogeneity of the studies, and considered I^2^ >75% as highly heterogeneous, we used random effect model if highly heterogeneous.

For mortality outcome, we used Peto’s odds ratio, as some studies showed zero mortality, so we were unable to calculate odds or risk ratios.

We used COVIDENCE online software which is used primarily for systematic reviews and meta-analysis for the screening process. We used Excel sheets for data extraction. We used Revman desktop version 5.4 for data analysis.

#### Subgroup analysis:

We did subgroup analysis of T1 only patients who did not receive mandatory pre or postoperative adjuvant chemo or radiotherapy.

#### Sensitivity Analysis:

1. We used HR and RR for the same outcome five-year OS and we found a difference between both statistical calculations.
2. We compared fixed effect model with random effect model: we didn’t find any change in the statistical direction of the outcomes, but the difference was slightly higher in the RE than the FE model.

#### Unit of analysis:

We planned to use data before the crossover, but there was no cross over trials in our finally included studies.

PICOTS and keywords used in the search.

**Table:** 

PICOTS:
Population: Patients with T1 and T2 rectal cancer
Intervention: minimal surgery
Comparative: standard surgery
Outcome:
• Primary outcomes: survival- cure- time to relapse
• Secondary outcomes: side effects- hospitalization days post operative
Time: Time to primary outcomes
Setting: Hospital for Surgery
**Search strategy key words:**
Total mesorectal excision OR Low anterior resection OR Abdominoperineal resection OR Laparoscopic OR Robotic OR Open Transanal TME
AND
Local Excision" OR Transanal Endoscopic Microsurgery OR Transanal Minimally Invasive Surgery" OR "Robotic transanal minimally invasive surgery OR Transanal Excision OR transanal endoscopic operation OR robotic transanal excision
AND
Rectal OR Anal
**Limitations for all:** Title and abstract

#### CENTRAL search:

#1 (Local Excision OR Transanal Endoscopic Microsurgery OR Transanal Minimally Invasive Surgery OR Robotic transanal minimally invasive surgery OR Transanal Excision OR transanal endoscopic operation OR robotic transanal excision) AND (Total mesorectal excision OR Low anterior resection OR Abdominoperineal resection OR Laparoscopic OR Robotic OR Open Transanal TME):ti,ab,kw AND (Rectal OR anal):ti,ab,kw (Word variations have been searched)

## Results:

- Our search retrieved 1243 studies.

- Central trials: 430
- Cochrane reviews: 11
- PubMed: 802

Two authors screened independently the title, abstracts, and full text articles using COVIDENCE online program. Any discrepancies between the authors were resolved through a consensus between them after discussion.

22 duplicates removed by Endnote.

- 1221 studies screened through COVIDENCE:
  21 duplicates removed by COVIDENCE
- 1200 studies (title and abstract) screened using COVIDENCE
  Exclusion after title and abstract screening: 1004 irrelevant
- 195 full texts were screened:
  Excluded after full text screening: 176
  **25** Wrong study design (including follow up of included studies)
  **21** abstracts only
  **68** Wrong patient population
  **18** Protocol only
  **12** non-English articles
  **10** clinical trials
  **6** reviews, editorial, commentary
  **7** Duplicate of included study
  **4** Wrong comparator
  **3** no data to include in the study
  **1** Wrong intervention
  **2** Wrong outcomes
- Included: 18 studies.

### Flow chart:

**Figure 1:**
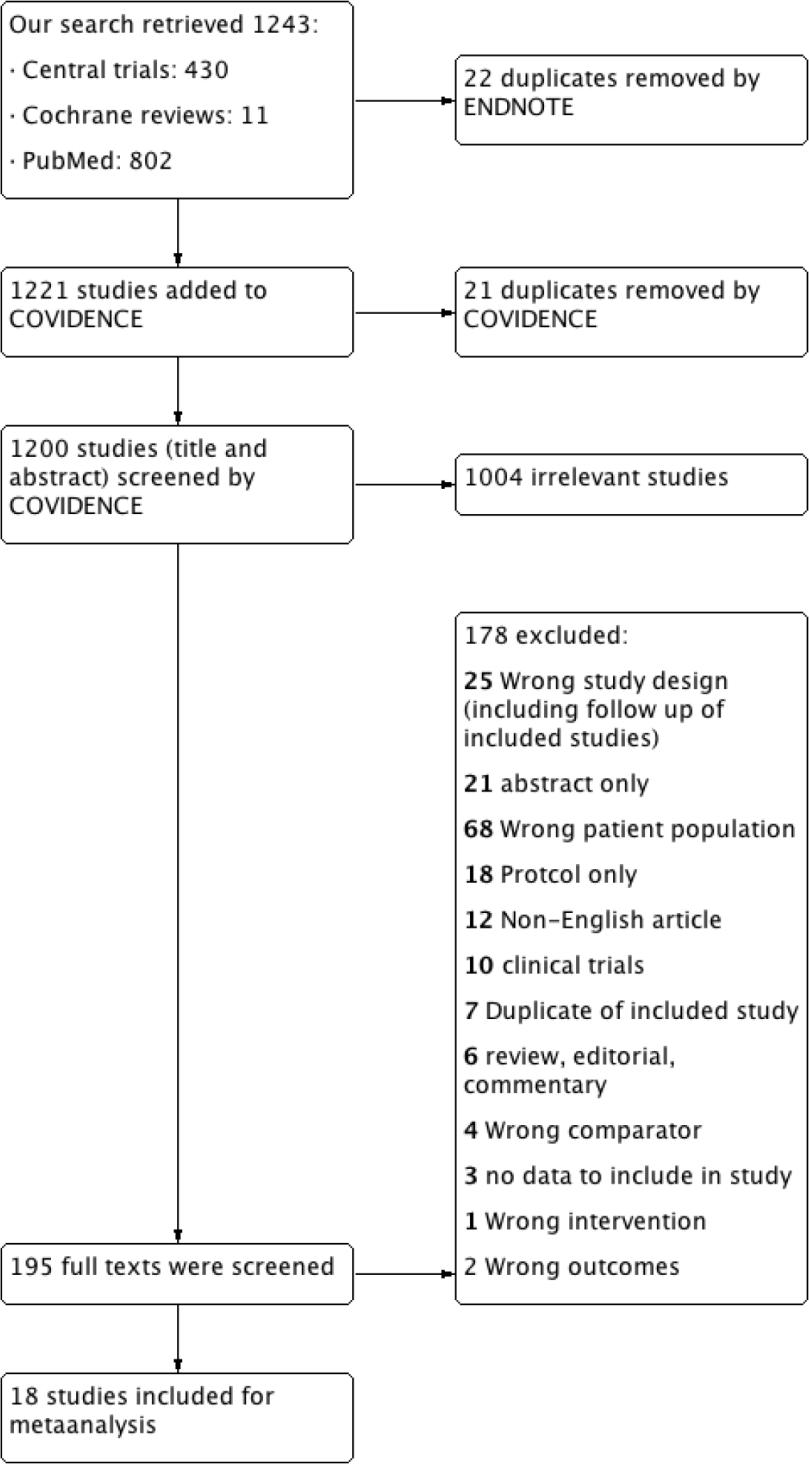
showing flow diagram of the meta-analysis.

We retrieved from the search strategy 1243 records. After entering the data in COVIDENCE, we excluded 21 duplicates. Endnote application desktop version X7.8 removed 22 duplicates. Then first two authors screened independently the titles and abstracts for exclusion of irrelevant records. Both authors screened independently 195 reports of studies in their full text for final inclusion. Any discrepancy or disagreements were resolved through a consensus with the principal investigator. (Figure 1)

We finally included 18 studies [7–24]. One of the included studies [9] had two cohorts; an RCT and a non-RCT, we treated the data of each study separately.

## Results:

We used random effect model due to clinical heterogeneity between studies (different surgical procedures, tumor staging, adjuvant chemo or radiotherapy). Six studies showed absent 30-days postoperative mortality in both groups, so we used peto-odds ratio. Postoperative mortality and morbidity were higher with RE than LE.

We did not find any difference in risk ratio (RR) between OS and DFS. But there were lower hazards ratios in OS and DFS with radical, as compared to local excision. Lower recurrence rate was associated with RE, as shown in table 1.

Four of the included studies were RCTs (Bach 2021 [9]; DeGraaf 2009 [10]; Lezoche 2012 [17]; Winde 1997 [23]).

Only one study reported the quality of life as an outcome for the interventions (endoluminal locoregional resection (ELRR) versus TME) in Leroche et al.[25]. We excluded this study because of absence of data on our primary or secondary assigned outcomes. We excluded Ellis et al. as the authors only reported the 7 years survival with no reported data on the 5-years OS or DFS [26].

From the included studies: nine were single center studies and nine were multicenter studies as shown in table 2.

### Forest plot for different outcomes:

#### Clinical trials registered on the topic:

It is shown in Supplementary table 1 that two trials are in the recruiting phase and two trials completed the recruiting of their designed number of patients but have not published their results yet. Two out of the four trials are open label with no blinding of participants or the physicians. Only one trial will restrict the population included to the T1N0 stage of rectal cancer and will recruit 326 patients in both arms. It will complete recruitment in May 2023.

## Discussion:

Previously, local excision was performed in patients who couldn’t undergo radical surgery. Due to high rate of survival and lower postoperative complications, it is now being used as a standard procedure for early rectal cancer. Though the patient is eligible for radical surgery, local excision is becoming the preferred technique for most patients [3].

In a retrospective population-based study of 2391 patients, there was no difference in disease specific survival between LE with RT vs APR, however overall survival was better in patients with APR for T2 disease [19]. As per Melnitchouk et al, in a study of 2084 patients with T1 tumors and 912 patients with T2 tumors, there was no difference in survival in patients who underwent LE and APR in T1 patients. Also, there was no difference in survival in T2 tumors between LE with chemotherapy and APR [18]. In a small sample study by Tokunaga et al, and colleagues, early rectal cancer patients who underwent chemoradiotherapy followed by LE were compared to TME. Disease-free survival and overall survival were not significantly different between the groups, however the study included only 5 patients in each cohort [27]. In a phase 2 trial of clinical stage T2N0 rectal cancer patients the three-year disease-free survival was 88% in the neoadjuvant chemotherapy followed by local excision compared to the radical excision group [28]. Currently prospective trials are being conducted for early distal rectal cancers comparing LE with neoadjuvant treatment to radical excision [17].

**Figure 2:**
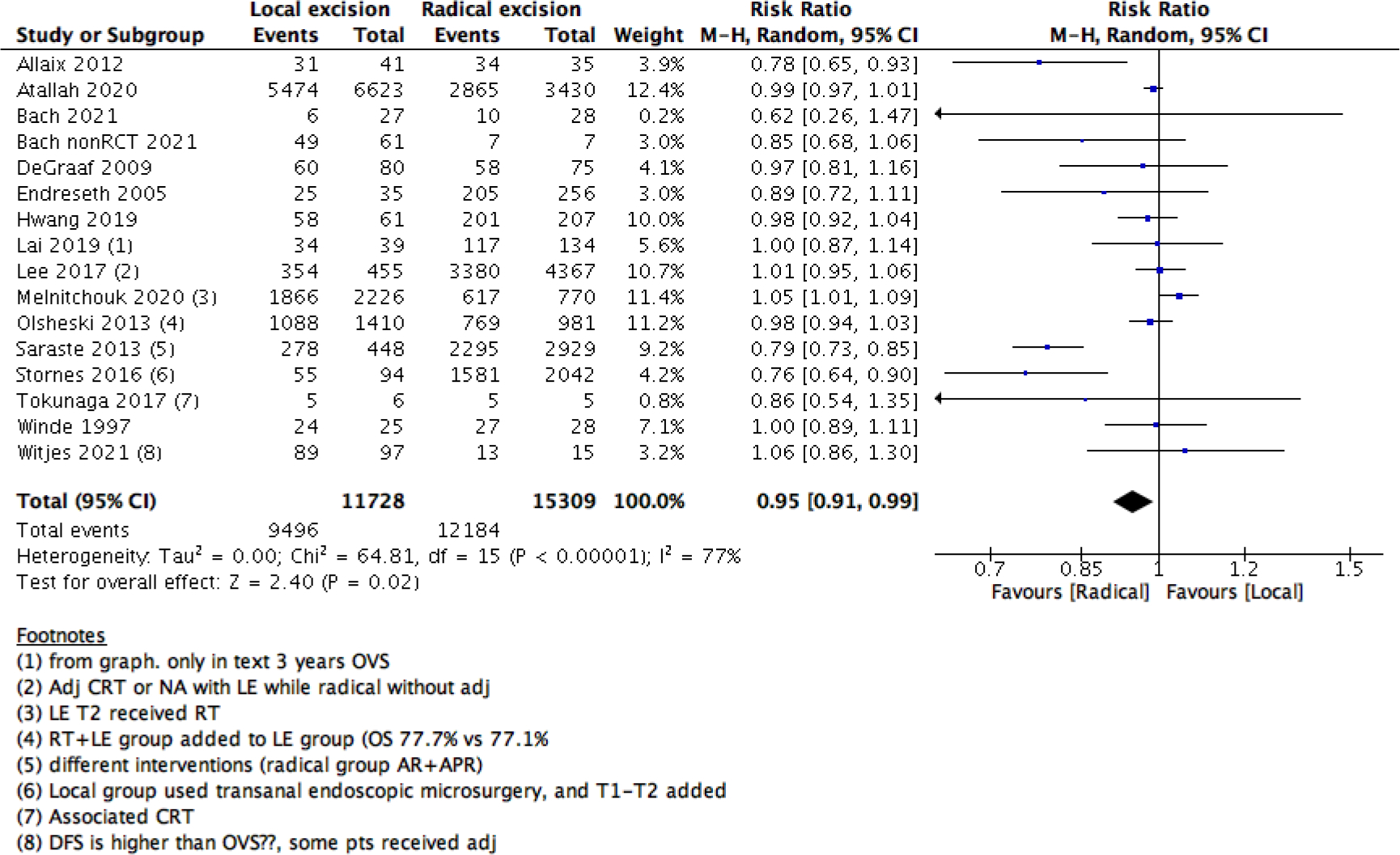
Forest plot of comparison: Survival analysis, outcome: Overall 5-year survival (Percentage).

**Figure 3:**
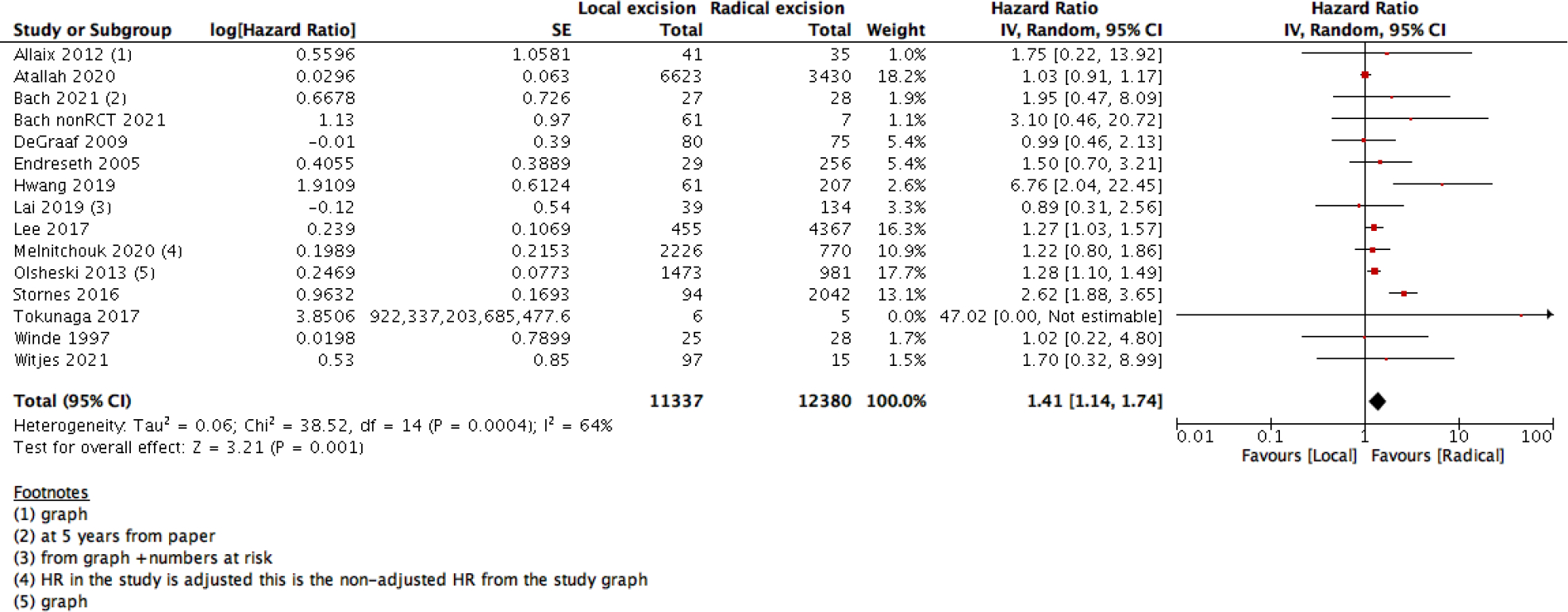
Forest plot of comparison: Survival analysis, outcome: overall 5-Year survival analysis (HR).

We measured survival analysis in two different statistical ways: hazards ratio depicting the decline of number of patients across time (5- years survival), and risk ratio depicting the difference between the number of patients at the start and end of the 5 years period. We found no difference in RR between LE versus RE (15 studies with 27037 participants; RR: 0.95; 95% CI: 0.91 to 0.99) (Figure 2). However, the HR showed a higher survival effect with RE as compared to LE (14 studies with 23717 participants; HR: 1.95, 95% CI: 1.36 to 2.78) (Figure 3). HR is a better indicator of the survival than RR.

**Figure 4:**
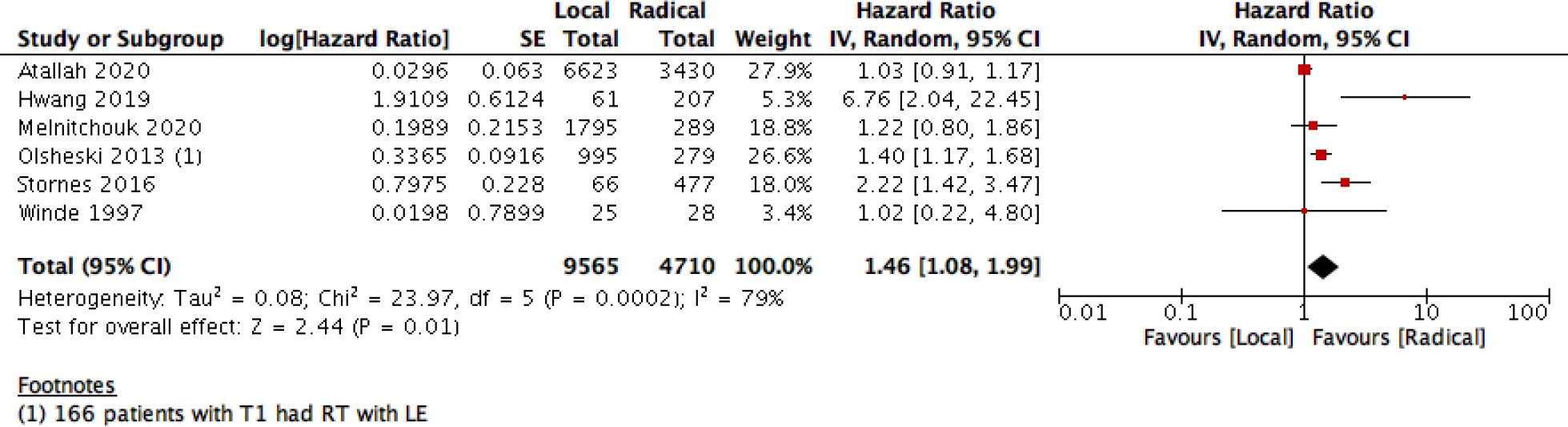
Forest plot of comparison (subgroup analysis): After removing adjuvant therapy and T2 patients, outcome: Overall survival T1 only- without CRT.

**Figure 5:**
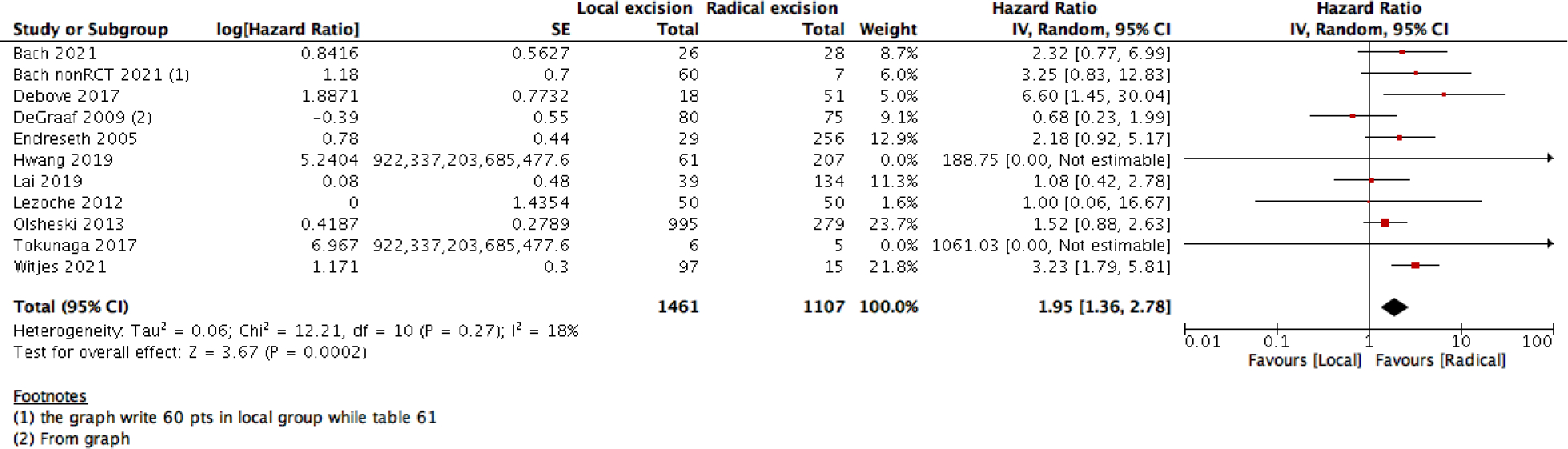
Forest plot of comparison: Survival analysis, outcome: Disease free survival HR.

**Figure 6:**
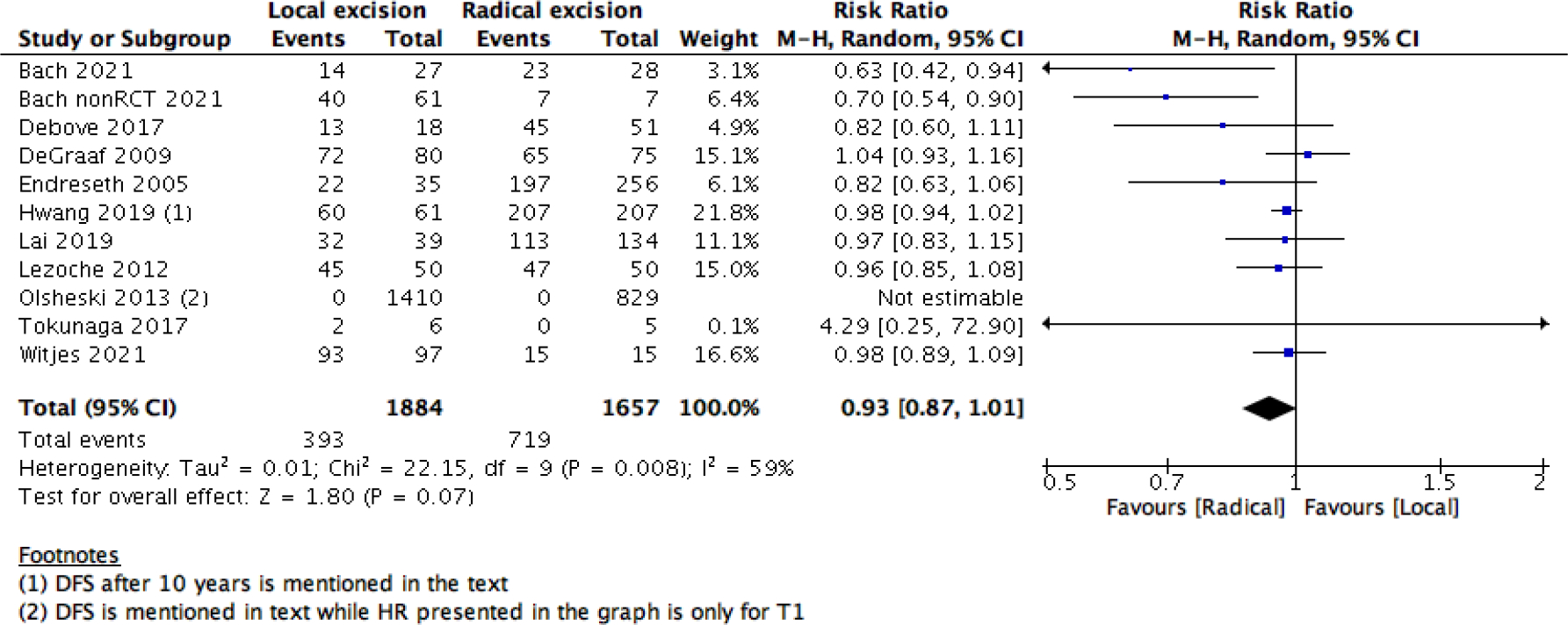
Forest Plot of comparison: survival analysis, outcome Disease free survival percentage RR.

This statistical preference of RE as opposed to LE maintained after subgroup analysis, removing all participants with T2 stage, or those who received CRT. We found that T1 rectal cancer patients show higher survival with RE than LE (6 studies with 14275 participants; HR 1.46; 95% CI 1.08– 1.99) (Figure 4). The same was found with DFS, as there was no difference between the two interventions when using the RR (10 studies with 3541participants; RR 0.93; 95% CI 0.87 to 1.01) (Figure 6). However, when using the HR for the same outcome we find that RE has higher survival than LE (10 studies with 2568 participants; HR 1.95; 95% CI 1.36 to 2.78) (Figure 5).

We found that early postoperative all-cause mortality (30 days postoperative) was higher in RE as compared to LE group (11 studies with 8800 participants; Peto’s OR 0.36; 95% CI 0.22– 0.59) (Supplementary Figure 1). This is consistent with the hazard of major surgery as opposed to a minor surgery.

A recent metaanalysis compared only TME to TEM surgical procedures for the outcome (local recurrence) and found only three RCTs for inclusion. They reported that there was no difference between the two interventions as regards to local recurrence or postoperative complications [29]. In our metaanalysis we included two of the three RCTs but unfortunately we didn’t find the full manuscript of Chen et al [30] to extract the data from, so it was not included on our final analysis.

The risk of local tumor recurrence was higher in LE than RE group (13 studies with 6952 participants; RR 2.85; 95% CI 1.86 to 4.36) (Supplementary Figure 2). This may raise the question, whether proceeding with a major surgery in early rectal cancer may benefit the patients more as it decreases the risk of recurrence and increase OS and DFS. Even though major surgeries are associated with more patient reservations, and higher risk of adverse events.

The number of days spent in the hospital in early postoperative stage were lower with LE than RE (5 studies with 3336 participants-standard mean difference −2.23 (95% CI: −3.64 to −0.83) (Supplementary Figure 3). The risk of early postoperative morbidity was higher with RE than LE group (7 studies with 671 participants; RR 0.31; 95% CI 0.22– 0.43) (Supplementary Figure 4). Both adverse events (postoperative hospitalization days and morbidity) and early postoperative 30 days mortality are consistent with the outcome of any major surgery, as RE is substantially carries more surgical risk and need more postoperative time for healing than the LE surgeries.

The techniques of the surgeries used in local excision in the included studies versus the techniques used the radical excision are mentioned in Supplementary table 2 in detail.

We found a Cochrane review on the topic [31] which measured the OS as a dichotomous outcome using odds ratio, and found similar results to our study. They found no difference between LE and TME. This concludes the fact that selecting the most appropriate statistical method could play a role in understanding the risk and benefit for the patients, but sometimes could be misleading.

A meta analysis including 29 RCTs with 6237 participants showed laparoscopic, robotic, and open TME had no difference in the intra and post operative morbidity, need for reoperation or presence of anastomotic leaks. However, the need for blood transfusion was lower with laparoscopic TME as compared to the other two. In addition, the risk of post operative infections and morbidity was lower with laparoscopic surgery as compare to open [32]. We didn’t compare between different kinds of surgical intervention in the same intervention group as this was beyond the scope of our study.

Atallah et al [8] carries the largest population weight in our study (51.3%), this may have an impact on our final results. It is a retrospective cohort- national study conducted in the United States using the National Cancer Database (NCDB). It is also included in our subgroup analysis as all patients in this study were T1 stage with no CRT.

## Conclusion:

LE for early-stage rectal cancer could decrease the risk of early postoperative mortality, hospitalization, and morbidity as compared to RE. However, RE could increase the 5-year OS, 5-year DFS and decrease the local recurrence. More large scale and national RCTs are needed to fully understand the benefit and risk of each of the surgical approaches. Currently a personalized approach to the patient’s condition is recommended to decide the best surgical and medical plan to the patient with early rectal cancer. The addition of chemo and radiotherapy for early rectal cancer needs further studies.

## Supplementary Material

1

## Figures and Tables

**Table 1:** showing the statistical results of the metaanalysis

Outcome or Subgroup	Number of Studies	Number of Participants	I^2^	Statistical methods	Estimate effect [CI]
1- Overall survival in 5-years (HR)	14	23717	64%	Hazard Ratio (IV, Random, 95% CI)	1.41 [1.14, 1.74]
2- Overall survival in 5-years (RR)	15	27037	77%	Risk Ratio (M-H, Random, 95% CI)	0.95 [0.91, 0.99]
3- Disease Free Survival in 5-years (HR)	10	2568	18%	Hazard Ratio (IV, Random, 95% CI)	1.95 [1.36, 2.78]
4- Disease Free Survival in 5-years (RR)	10	3541	59%	Risk Ratio (M-H, Random, 95% CI)	0.93 [0.87, 1.01]
5- Local recurrence rate	13	6952	40%	Risk Ratio (M-H, Random, 95% CI)	2.85 [1.86, 4.36]
6- All-cause mortality in 30-days	10	8800	13%	Peto Odds Ratio (Peto, Fixed, 95% CI)	0.36 [0.22, 0.59]
7- Total post-operative morbidity	7	671	71%	Risk Ratio (M-H, Random, 95% CI)	0.38 [0.18, 0.80]
8- Hospital stay post-operative	5	3336	99%	Std. Mean Difference (IV, Random, 95% CI)	−2.23 [−3.64, −0.83]
9- *Subgroup analysis:* Overall survival T1 only- without obligatory CRT	6	14275	79%	Hazard Ratio (IV, Random, 95% CI)	1.46 [1.08, 1.99]

**Table 2:** showing the characteristics of the included studies

Study ID	Stage of the tumor	Number of patients in the study	Age of the patients	Number of centers	Country of the conducted study
Olsheski 2013	T1-T2 N0M0	2391	APR 68 vs LE alone 68 vs LE+RT 67	Multicenteral	USA
Tokunaga 2017	T1T2	11	63±11 (TLE) 65±13 (47–76) (TME)	Single center	Japan
Saraste 2013	T1T2N0M0	3694	median age AR 70 y vs APR 71y vs LE 75 y	Multicentral	Sweden
Melnitchouk 2020	T1T2	2996		Multicentral	USA
Stornes 2016	T1T2	2136	T1: (TEM)72.5 vs 68.2 (TME); T2: 81.7 vs 69.9	Multicentral	Norway
Witjes 2021	T1T2N0M0	112	72 (EMR), 67 (TEM/TAMIS), 62 (LAR/APR)	Multicentral	UK
Allaix 2012	T2	78	72 (38–91) TEM vs 65 (34–90) LR	Single center	Italy
Atallah 2020	T1	10053	Multiple groups (<55, 55–64, 65–74, >75)	Multicentral	USA
Bach 2021	T1T2	55 RCT; 68 Non RCT	65 (52–79) LE vs 65 (49–83) RR	Multicentral	UK
DeGraaf 2009	T1	155	TEM 71 (44–92) vs TME 67(48–83)	Single center	Netherlands
Debove 2017	T1	91	62 +/- 12	Single center	France
Elmessiry 2014	T1T2	153	68.7 +/-11.9 LE vs 65.3 +/-15.3 TME	Single center	USA
Endreseth 2005	T1T2	291	68 TME vs 77 LE	Single center	Norway
Hwang 2019	T1	268	58.0 ± 9.5 TAE vs 59.0 ± 9.6 TME	Single center	Korea
Lai 2019	T1T2	173	59.7 ± 13.9 LE vs 63.0 ± 12.9 TME	Single center	Taiwan
Lee 2017	T2	4822	65.6 LE+Neoadj CRT vs 65.9 LE+ Adj CRT vs 66 RR	Multicentral	USA
Lezoche 2012	T2N0M0	100	ELRR 66 vs 66 TME	Multicentral	Italy
Winde 1997	T1	54		Single center	Germany

## Data Availability

Data can be obtained if requested from the first author of the article.

## List of abbreviations:

CI: Confidence Interval
CRC: colorectal cancer
CRT: Chemoradiotherapy
DFS: Disease Free Survival
HR: Hazards Ratio
LAR: Low Anterior Resection
LE: Local Excision
OVS: Overall Survival
RCTs: Randomized Controlled Trials
RE: Radical Excision
RR: Risk Ratio
RT: Radiation Therapy
TAMIS: Transanal Minimally Invasive Surgery
TEM: Transanal Endoscopic Microsurgery
TME: Total Mesorectal Excision
