# Supplementary material for: Radical versus Local Surgical Excision for Early Rectal Cancer: A Systematic Review and Meta-Analysis
Source: Arch Intern Med Res. Author manuscript; Available in PMC 2024 Apr 11. (PMC11008054; doi:10.26502/aimr.0160)
Supplement: 1 [file NIHMS1961193-supplement-1.pdf]

Supplementary data

Study or Subgroup	Local excision Events	Local excision Total	Radical excision Events	Radical excision Total	Weight	Peto Odds Ratio Peto, Fixed, 95% CI
Allaix 2012	0	41	0	35		Not estimable
Bach 2021	0	27	0	28		Not estimable
Bach nonRCT 2021	0	61	0	7		Not estimable
DeGraaf 2009	0	80	3	75	4.8%	0.12 [0.01, 1.20]
Endreseth 2005	1	35	6	256	4.7%	1.24 [0.12, 12.42]
Lai 2019 (1)	2	39	25	134	25.9%	0.36 [0.14, 0.96]
Lee 2017	3	455	64	4367	36.5%	0.56 [0.24, 1.27]
Lezoche 2012 (2)	0	50	0	50		Not estimable
Melnitchouk 2020 (3)	10	2226	13	770	28.2%	0.20 [0.08, 0.50]
Tokunaga 2017	0	6	0	5		Not estimable
Winde 1997	0	25	0	28		Not estimable
**Total (95% CI)**		**3045**		**5755**	**100.0%**	**0.36 [0.22, 0.59]**
Total events	16		111			

Heterogeneity: $Chi^2 = 4.62$, df = 4 (P = 0.33); $I^2 = 13\%$

Test for overall effect: Z = 4.04 (P < 0.0001)

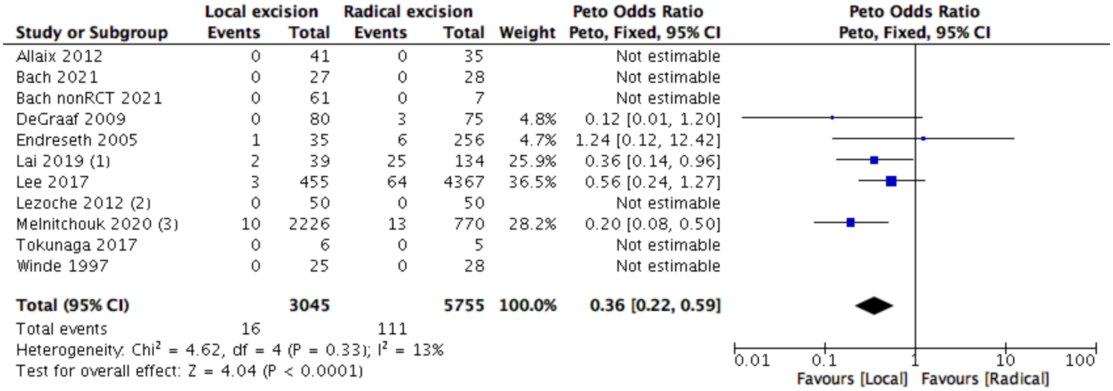

Footnotes

(1) early post operative

(2) no mortality

(3) 30 days post op

Supplementary Figure 1: Forest plot of comparison: Survival analysis, outcome: Recurrence

Study or Subgroup	Local excision Events	Local excision Total	Radical excision Events	Radical excision Total	Weight	Risk Ratio M-H, Random, 95% CI
Allaix 2012	8	41	3	35	7.9%	2.28 [0.65, 7.93]
Bach 2021	3	27	0	28	1.9%	7.25 [0.39, 134.07]
Bach nonRCT 2021	10	61	0	7	2.2%	2.71 [0.17, 41.96]
DeGraaf 2009	19	80	0	75	2.1%	36.59 [2.25, 595.53]
Elmessiry 2014	11	38	3	39	8.3%	3.76 [1.14, 12.44]
Endreseth 2005	2	35	31	256	6.8%	0.47 [0.12, 1.89]
Hwang 2019 (1)	8	61	4	207	8.6%	6.79 [2.12, 21.77]
Lai 2019	5	39	11	134	10.4%	1.56 [0.58, 4.22]
Lezoche 2012	4	50	3	50	6.4%	1.33 [0.31, 5.65]
Saraste 2013 (2)	50	448	77	2929	21.1%	4.25 [3.02, 5.98]
Stornes 2016	13	94	76	2042	17.3%	3.72 [2.14, 6.45]
Tokunaga 2017	2	6	0	5	2.1%	4.29 [0.25, 72.90]
Winde 1997	1	25	0	28	1.7%	3.35 [0.14, 78.60]
Witjes 2021	3	97	1	15	3.2%	0.46 [0.05, 4.17]
**Total (95% CI)**		**1102**		**5850**	**100.0%**	**2.85 [1.86, 4.36]**
Total events	139		209			

Heterogeneity: $Tau^2 = 0.19$; $Chi^2 = 21.52$, df = 13 (P = 0.06); $I^2 = 40\%$

Test for overall effect: Z = 4.84 (P < 0.00001)

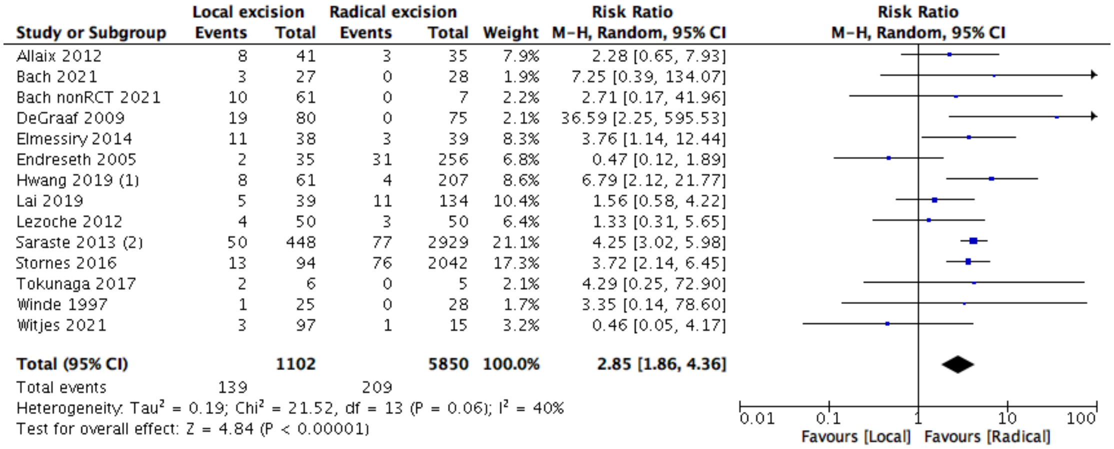

Footnotes

(1) systemic and local recurrence after variable periods

(2) Both AR(1947)+APR (982) added in radical group

Supplementary Figure 2: Forest plot of comparison: Adverse events, outcome early post-operative morbidity

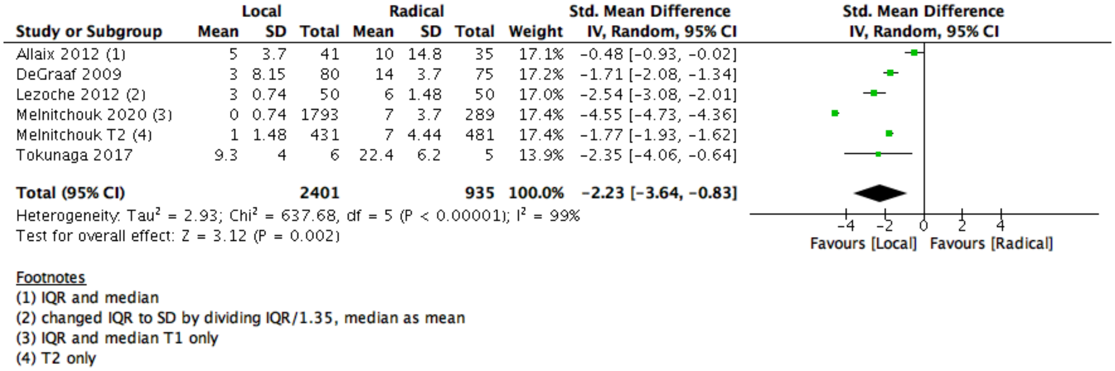

Supplementary Figure 3: Forest plot of comparison: Adverse events, outcome: Hospital Stay

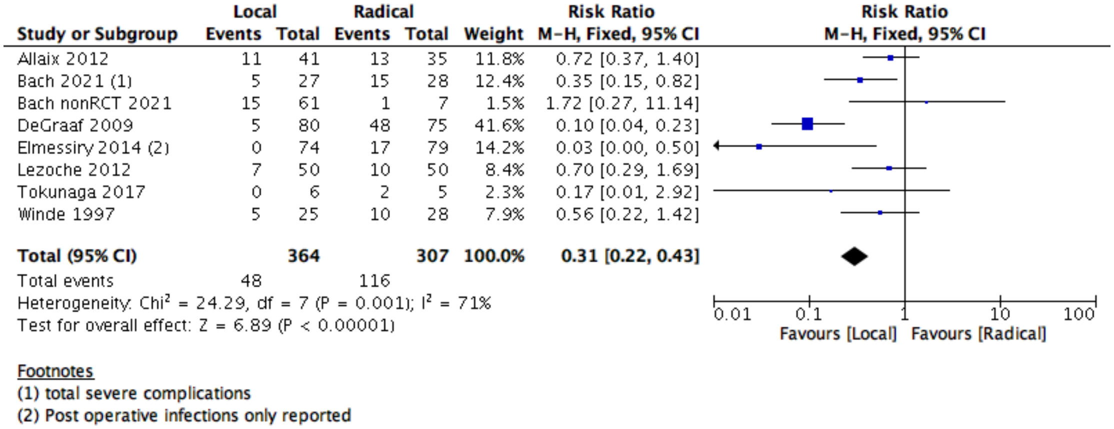

Supplementary Figure 4: Forest plot of comparison: Survival analysis, outcome: EARLY Post-operative Morbidity

**Status**	**Registration number**	**Type of surgery**	**Type of the study**	**Start date**	**Expected finish date**	**Total No. of patients**	**Stage of the tumor**
Completed -	NCT01308190	transanal microsurgery+CR vs	RCT	8(2010)	7(2021)	173	T2-T3

unpublished		TME					
Completed - unpublished	NCT02550769 (NOTESvsL-LAR)	Trans anal TME vs Laparoscopic-LAR	RCT (open label)	4(2015)	6(2021)	116	cT1-2-3, cN0-1, cM0
Recruiting	NCT03548844	Local excision vs TME	RCT (open label)	5(2018)	5(2023)	326	T1 N0
Recruiting	NCT04098471	Transanal LE with and without radiotherapy vs TME	RCT	12(2019)	12(2026)	300	T2N0M0

Supplementary Table 1: show ongoing and finished clinical trials on ClinicalTrials.gov

**Study ID**	**Surgeries performed**
Olsheski 2013	LE versus APR
Tokunaga 2017	LE vs TME
Saraste 2013	LE, AR, APR, Hartman?
Melnitchouk 2020	LE versus APR
Stornes 2016	TEM vs TME
Witjes 2021	TEM; TAMIS; ESD; EMR vs APR ; TME
Allaix 2012	TEM vs RT + TEM vs LR
Atallah 2020	LE (open or laparoscopic) vs RR
Bach 2021	RT followed by TEM vs LAR
DeGraaf 2009	RT before TEM vs TME alone
Debove 2017	LE vs TME vs LE+TME
Elmessiry 2014	TME vs LE
Endreseth 2005	Transanal Excicison vs Major Surgery (AR,APR,Hartmann)
Hwang 2019	TAE vs TME (some patients received chemo and radio)
Lai 2019	LE vs TME
Lee 2017	NA-CRT+LE vs LE+adj CRT vs RS
Lezoche 2012	Endoluminal locoregional resection (ELRR) by TEM vs laparoscopic TME (All patients had chemoradiotherapy)
Winde 1997	TEM vs AR TME

Supplementary Table 2: showing the surgical procedures
